# Geo-mapping of caries and obesity in preschool children: a Swedish register-based study

**DOI:** 10.1186/s12903-026-07783-z

**Published:** 2026-01-30

**Authors:** Lucy Zhou, Daniel Åberg, Qinyun Lin, Carl Bonander, Ulf Strömberg

**Affiliations:** 1 University of Gothenburg, School of Public Health and Community Medicine, Institute of Medicine, Box 469, Gothenburg, 405 30 Sweden; 2 Region Kronoberg, Department of Monitoring and Quality Assessment, Växjö, Sweden; 3 Region Kronoberg, Department of Research, Växjö, Sweden

**Keywords:** Caries, Obesity, Early childhood, Health disparity, Prevention, Spatial modelling, Neighborhood deprivation

## Abstract

**Background:**

Lifestyle interventions addressing both oral health and obesity in childhood have attracted attention due to the shared risk factors. Prioritization of resources to lifestyle interventions within pediatric dental care should rely on a rational basis. We consider a dental care setting where geographically targeted interventions could be pursued, and develop an analytic approach, geo-mapping, to identify high-risk neighborhoods.

**Methods:**

The population of interest comprised the children between 3 and 6 years old who resided in a Swedish region, Kronoberg (total population of 204,000), at the end of 2024. We used register data provided by all dental clinics and child health centers in the Kronoberg region. The binary outcomes caries (dfs > 0) and obesity (according to the internationally recommended classification) were analyzed combined. Multinomial outcome data aggregated at neighborhood-level (totally, 112 neighborhoods) were analyzed by Bayesian spatial modelling, with adjustments for sex and age. Furthermore, we estimated associations with neighborhood deprivation, classified according to national quintiles Q1 (least deprived) to Q5 (most deprived).

**Results:**

The study population included 8,293 children (88% coverage), 888 of whom (10.7%) were assessed with caries, 226 (2.7%) with obesity, and 29 (0.3%) with coexisting caries and obesity. We found no statistical evidence for an individual-level association between caries and obesity; the prevalence-odds ratio of obesity for children with vs. without caries was estimated to 1.24, with a 95% confidence interval ranging from 0.83 to 1.84. Twenty-four neighborhoods were identified as having an elevated prevalence of children with caries (posterior probability > 0.80); comprising 1,960 children, 447 of whom (22.8%) were assessed with caries. Five of these 24 neighborhoods coincide with 5 of the 10 neighborhoods identified as having an elevated prevalence of obese children. The estimated association with neighborhood deprivation was more pronounced for caries (odds ratio Q5 vs. Q1 = 6.28; 95% credible interval [CrI]: 4.23–9.34) than for obesity (1.97; 95% CrI: 1.15–3.38).

**Conclusions:**

Even though this analysis yielded a rational basis for prioritizing resources to preventive measures focusing on oral health in early childhood – to providers of dental care for preschool children predominantly living in the most deprived neighborhoods – we found no support for a concurrent prioritization of more comprehensive lifestyle interventions, addressing both caries and obesity.

**Clinical trial number:**

Not applicable.

**Supplementary Information:**

The online version contains supplementary material available at 10.1186/s12903-026-07783-z.

## Background

Dental caries and obesity are two major adverse health conditions in early childhood that bring to the fore preventive measures for children’s future health. Caries experience during early childhood is the single strongest predictor of caries experience in adolescence and into adult [1]. There is also substantial evidence that obesity or overweight in childhood predicts obesity in adolescence and adulthood [2]. It has been inferred that preventive efforts among children at high risk of obesity should start before 5–7 years of age [3], but targeting solely obese or overweight preschool children needs to be considered carefully as this may not essentially reduce the overall burden of adult obesity [2, 3].

Lifestyle interventions addressing both oral health and obesity in childhood have attracted attention due to the shared risk factors, particularly dietary habits [4]. Integrating lifestyle interventions into pediatric dental care settings has the potential to reach high-risk children and address health disparities. Socioeconomically linked disparities in childhood caries and obesity have been extensively observed [5, 6], which calls for geographically targeted interventions – targeting deprived neighborhoods. To form a rational basis for targeting high-risk neighborhoods, the geographic pattern of caries and obesity/overweight among children should be examined. At the individual level, a positive correlation between caries and obesity in early childhood (< 7 years of age) has been indicated by meta-analyses, although the quality of evidence extracted from previous studies varied considerably [7, 8].

An analytic method for targeting neighborhoods where the living children have relatively high caries rates has been launched in this Journal [9]. The presented application of that method, referred to as geo-mapping, formed a basis for reallocating tax-funded financial resources to the dental care clinics within a Swedish region (Halland) – for tailoring supportive and preventive measures to children on account of their caries status [10]. Geo-mapping was shown to identify geographic patterns tied to sociodemographic factors [9]. Geo-mapping also has the potential to identify geographic patterns tied to environmental factors, such as fluoride levels in drinking water [11].

In this paper, we raise the question whether targeting of high-risk neighborhoods – considering both caries and obesity/overweight in early childhood – can provide a rational basis for prioritizing resources to comprehensive lifestyle interventions within pediatric dental care. Specifically, we develop the geo-mapping approach for this purpose and present an application for a Swedish region, Kronoberg.

## Methods

### Study population

The population of interest comprised the children between 3 and 6 years old who resided in the Kronoberg region at the end of 2024. The Kronoberg region has a total population of 204,000 and is located adjacently east of the south-western Swedish region Halland (population of 345,000), where the geographic pattern of childhood caries has been studied previously [9].

The healthcare organization in Sweden is regionalized [12]. In the Kronberg region (one of the 21 regions in Sweden) there are 12 public and 32 private general dental care clinics proving free dental care to children, and 22 public and 10 private child health centers providing free health checks and development monitoring for children up to age 6. The Kronoberg region has implemented a capitation model in which tax-funded money is allocated to each general dental care clinic proportionally to the number of listed children, where 80% of the money is a direct proportion and the remaining 20% is a weighted proportion according to the Care Need Index (CNI) [13].

### Outcome data

The healthcare registers for the Kronoberg region include comprehensive data from both public and private clinics. For the population of interest, we retrieved pseudonymized personal data on caries and body mass index (BMI), most recently assessed by the healthcare providers. In other words, we retrieved outcome data from the children’s last visits at any of the 44 general dental clinics (caries) and any of 32 child health care centers (BMI) located in the Kronoberg region. Caries was assessed as dfs > 0. Obesity and overweight including obesity were defined according to ISO-BMI, i.e., the International Obesity Task Force (IOTF) body mass index (BMI) categorization based on age- and sex-specific BMI thresholds [14]. The dates of the caries and BMI assessments were also retrieved.

### Sociodemographic data

Individual-level data on birth year, sex, and residential neighborhood were obtained from the registries. The neighborhoods correspond to Statistics Sweden’s small-area division of Sweden referred to as Demographic Statistical Areas (*Demografiska statistikområden* in Swedish), launched in 2018 to facilitate the monitoring of segregation and socioeconomic conditions [15]. The Kronoberg region is divided into 112 such neighborhoods; the number of inhabitants varies between 700 and 3,500, with a median of 1,800 people.

We considered a neighborhood-level covariate: economic standard, defined as the proportion of inhabitants with low household income – corresponding to the 1 st quartile of the household income distribution for all residents of Sweden – out of all inhabitants of a neighborhood. Economic standard has been suggested as an appropriate neighborhood-level deprivation indicator for Sweden, and it correlates strongly with other proposed indices of multiple deprivation [16, 17]. This covariate is referred to as neighborhood deprivation in the following. Neighborhood deprivation was categorized into national quintiles Q1–Q5 (Q1 = 20% of the 5,984 neighborhoods in Sweden with the lowest proportions [least deprived], and so on to Q5 [most deprived]).

### Statistical methods

Outcome data were analyzed combined. Primarily, we considered the following four outcome categories: {no caries, no obesity}, {caries, no obesity}, {no caries, obesity}, and {caries, obesity}. We also analyzed the multinomial outcome variable {no caries, no overweight or obesity}, {caries, no overweight or obesity}, {no caries, overweight including obesity}, and {caries, overweight including obesity}. The individual-level association between caries and obesity/overweight was estimated by the odds ratio (OR) comparing the odds of obesity (or overweight including obesity) between the children with and without caries, along with the 95% confidence interval (CI).

For the geo-mapping of the neighborhood-level outcome data, we employed a Bayesian model where spatially structured random effects were modelled using an intrinsic conditional autoregressive prior (specifically, the model referred to as BYM2 [18]). The model was estimated using Integrated Nested Laplace Approximation (INLA) via the R-INLA package [19] (R version 4.4.3), with priors set to the package defaults (the default penalized complexity prior for the marginal precision τ_b_ [pc.prec with pc.u = 1 and pc.alpha = 0.01] and the default prior for the mixing parameter φ [pc with pc.u = 0.5 and pc.alpha ≈ 2/3]). Neighborhood adjacency was defined using queen contiguity based on the small-area polygons, where areas sharing a border or a vertex are neighbors. As R-INLA does not natively support multinomial likelihoods, the multinomial outcome model was fitted using the Poisson trick [20]. Sex and age were incorporated as fixed-effect covariates. Age was categorized based on birth year: 3–4 years old (birth year 2020–2021), 5 years old (birth year 2019), and 6 years old (birth year 2018). Ages 3–4 years were combined due to low numbers assessed with caries among the youngest children.

To assess model adequacy, we conducted a posterior predictive check, comparing observed neighborhood-level prevalences with posterior predictive medians along with 95% posterior predictive intervals (corresponding to the 95% credible intervals [CrIs] for the model-based prevalences).

The geo-mapping results are presented based on each neighborhood’s posterior probability of the prevalence for a specific adverse outcome – {caries, no obesity}, {no caries, obesity}, or {caries, obesity} (and analogously for overweight including obesity instead of obesity) – exceeding the corresponding prevalence in the whole region, i.e., in the total study population. To calculate these probabilities, we repeatedly sampled 1,000 times from the posterior distributions of the fitted model. In each posterior sample, we normalized the estimated probabilities (exponentiated linear predictions) of each outcome category to sum to one. Based on these posterior probability calculations, the neighborhoods with an elevated prevalence of a specific adverse outcome (compared to the regional average) were identified, using 0.80 as the cut-off for the posterior probability, which has been found to be a reasonable trade-off between false-positive and false-negative rates in two independent simulation studies [21, 22].

Associations of each adverse outcome category with neighborhood deprivation were estimated by adding the deprivation covariate (Q1 [least deprived] to Q5 [most deprived]) to the geo-mapping model, which yielded the fixed-effect estimates with 95% CrIs.

## Results

### Individual-level data

The registry holder identified a population of 8,810 children born between 2018–2021 who were registered as having at least one caries assessment at a dental clinic in the Kronoberg region by the end of November 2024. It was recognized that 340 children had moved out of the Kronoberg region, based on the information on their current addresses at the time. The remaining population of 8,470 covered 90% of the total population of 3–6-year-olds living in the Kronoberg region per December 31 st, 2024 (*N* = 9,429). Furthermore, 70 children with protected identity and 107 children with incomplete or inadequate BMI data (*n* = 93 with missing or incorrectly registered weight/height and *n* = 14 who were younger than 24 months at their most recent child health center visit) were excluded, yielding a study population of 8,293.

The median time difference between the outcome assessments (derived from each child’s most recent dental clinic visit [caries] date minus the date of the most recent child health care visit [BMI]), was −0.08 months (25th to 75th percentile: −6.2–3.6; minimum to maximum: −35.0–28.3). For 914 children (11%), the two outcomes were assessed more than one year apart.

In the study population, 888 (10.7%) children were assessed with caries, 226 (2.7%) with obesity, and 1,070 (12.9%) with overweight including obesity. Twenty-nine children (0.3%) were assessed with coexisting caries and obesity, and 113 children (1.4%) with coexisting caries and overweight including obesity. There was no statistical evidence for an overall individual-level association between caries and obesity (OR = 1.24; 95% CI: 0.83 to 1.84), nor between caries and overweight including obesity (OR = 0.98; 95% CI: 0.80 to 1.21).

Table 1 shows the outcome distribution for caries and obesity by sex and birth year. As expected, the prevalence of caries increased by age. More girls than boys were assessed with obesity. Supplementary Table S1 shows the corresponding outcome distributions for caries and overweight including obesity.

**Table 1:** Description of the study population and the outcomes of caries and obesity

		**No. of children**	**Children with outcome {no caries, no obesity}, n (%)**	**Children with outcome {caries, no obesity}, n (%)**		**Children with outcome {no caries, obesity}, n (%)**	**Children with outcome {caries, obesity}, n (%)**
	Study population	8,293	7,208 (86.9%)	859 (10.4%)	197 (2.4%)		29 (0.3%)
Sex	Boys	4,278	3,734 (87.3%)	453 (10.6%)	77 (1.8%)		14 (0.3%)
	Girls	4,015	3,474 (86.5%)	406 (10.1%)	120 (3.0%)		15 (0.4%)
Age (years)	6	2,217	1,778 (80.2%)	373 (16.8%)	50 (2.3%)		16 (0.7%)
	5	2,295	1,962 (85.5%)	270 (11.8%)	57 (2.5%)		6 (0.3%)
	3–4	3,781	3,468 (91.7%)	216 (5.7%)	90 (2.4%)		7 (0.2%)

### Geo-mapping results

**Fig. 1:**
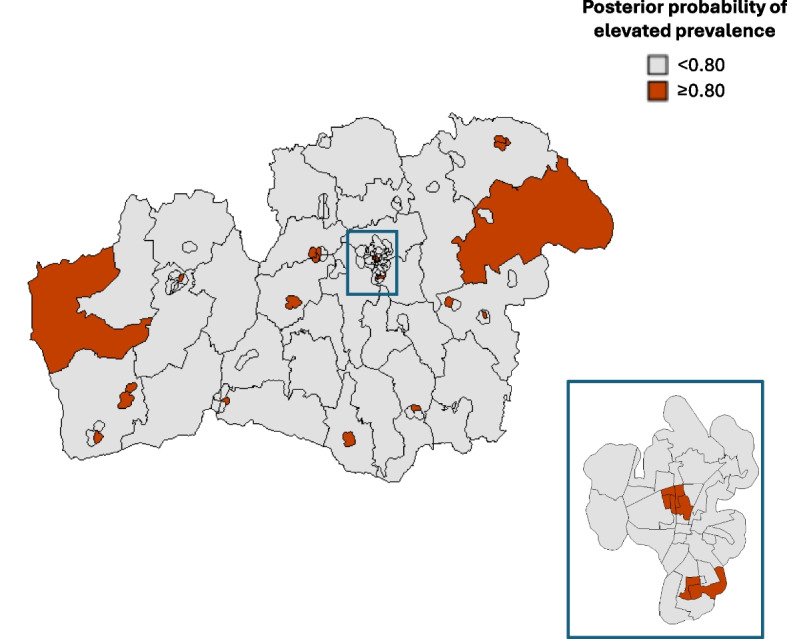
Geographic variation in the prevalence of caries among 3–6-year-old children. The map of the study region (Kronoberg, Sweden) is divided into 112 neighborhoods and visualizes, in dark red color, the 24 neighborhoods with elevated prevalence (posterior probability > 0.80). One urban area (Växjö) is shown in an enlarged, separated map. The posterior probabilities were derived from the Bayesian spatial model fitted to the neighborhood-level multinomial outcome data, including sex and age as fixed-effect covariates

**Fig. 2:**
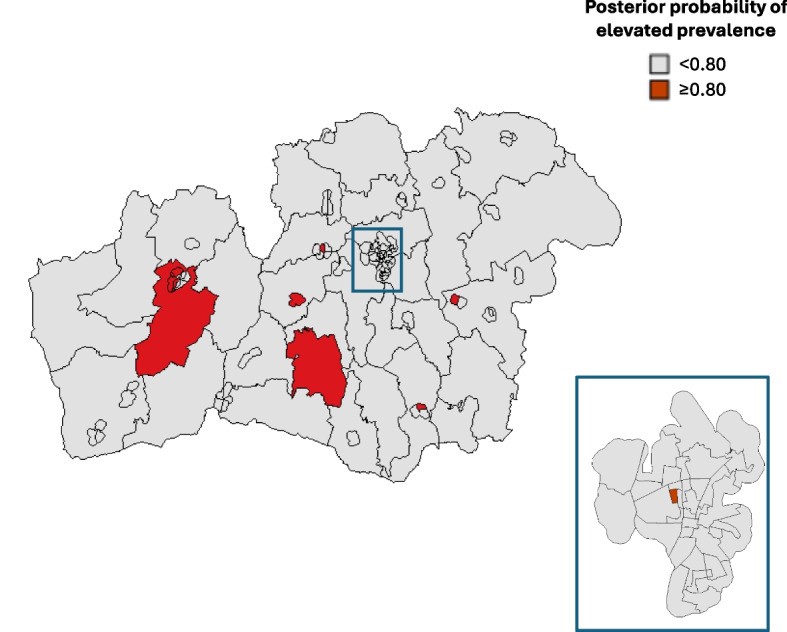
Geographic variation in the prevalence of obesity among 3–6-year-old children. The map of the study region (Kronoberg, Sweden) is divided into 112 neighborhoods and visualizes, in dark red color, the 10 neighborhoods with elevated prevalence (posterior probability > 0.80). One urban area (Växjö) is shown in an enlarged, separated map. The posterior probabilities were derived from the Bayesian spatial model fitted to the neighborhood-level multinomial outcome data, including sex and age as fixed-effect covariates

The geo-mapping of the primary multinomial outcome ({no caries, no obesity}, {caries, no obesity}, {no caries, obesity}, and {caries, obesity}) identified 24 neighborhoods as having an elevated prevalence of children with caries (posterior probability > 0.80) (Fig. 1). These 24 neighborhoods comprised 1,960 children, 447 of whom (22.8%) were assessed with caries. Ten neighborhoods were identified as having an elevated prevalence of obese children (Fig. 2); comprising 788 children, 50 of whom (6.3%) were obese. Five of the 24 “high-risk-caries” neighborhoods coincide with 5 of the 10 “high-risk-obesity” neighborhoods. These 5 coinciding neighborhoods were identified as having an elevated prevalence of children with coexisting caries and obesity.

The posterior predictive check is presented in Supplementary Figure S1.

The geo-mapping of the secondary multinomial outcome, considering overweight including obesity, identified 24 neighborhoods as having an elevated prevalence children with overweight including obesity. Sixteen of these 24 neighborhoods showed an elevated prevalence of children with coexisting caries and overweight including obesity.

### Associations with neighborhood deprivation

Table 2 shows the primary outcome distribution by neighborhood deprivation. The corresponding outcome distributions for caries and overweight including obesity are shown in Supplementary Table S2. These crude data clearly indicate higher prevalences of early childhood caries with higher degree of neighborhood deprivation.

**Table 2:** Prevalences of caries and obesity in groups of children according to neighborhood deprivation

Neighborhood deprivation* (number of neighborhoods)	No. of children	Children with outcome {no caries, no obesity}, n (%)	Children with outcome {caries, no obesity}, n (%)	Children with outcome,{no caries, obesity}, n (%)	Children with outcome {caries, obesity}, n (%)
Q1 (13)	1,220	1,145 (93.9%)	51 (4.2%)	23 (1.9%)	1 (0.1%)
Q2 (17)	1,373	1,281 (93.3%)	67 (4.9%)	24 (1.7%)	1 (0.1%)
Q3 (24)	1,786	1,613 (90.3%)	118 (6.6%)	50 (2.8%)	5 (0.3%)
Q4 (30)	1,921	1,653 (86.0%)	227 (11.8%)	40 (2.1%)	1 (0.1%)
Q5 (28)	1,993	1,516 (76.1%)	396 (19.9%)	60 (3.0%)	21 (1.1%)

^*^Q1 = least deprived to Q5 = most deprived

The model yielded the adjusted estimates of the associations between each adverse outcome category and neighborhood deprivation. The estimates revealed a more pronounced association for the outcome {caries, no obesity} (OR Q5 vs. Q1 = 6.28; 95% CrI: 4.23 to 9.34) than for {no caries, obesity} (OR Q5 vs. Q1 = 1.97; 95% CrI: 1.15 to 3.38) (Table 3). As the outcome {caries, obesity} was rarely observed, we obtained imprecise association estimates across Q1–Q5 (Table 3). Twenty-one of the 29 children with coexisting caries and obesity lived in the most deprived neighborhoods (Table 2), which yielded a markedly strong, but extremely imprecisely estimated, association for the outcome {caries, obesity} (OR Q5 vs. Q1 = 26.2; 95% CrI: 3.51 to 195) (Table 3).

**Table 3:** Estimated associations of caries and obesity with neighborhood deprivation

Neighborhood deprivation*	OR (95% CrI) for outcome {caries, no obesity}	OR (95% CrI) for outcome {no caries, obesity}	OR (95% CrI) for outcome {caries, obesity}
Q1	1.00 (reference)	1.00 (reference)	1.00 (reference)
Q2	1.20 (0.76–1.91)	0.91 (0.49–1.70)	0.89 (0.06–14.2)
Q3	1.78 (1.16–2.74)	1.48 (0.84–2.58)	5.42 (0.63–46.5)
Q4	3.24 (2.17–4.85)	1.14 (0.64–2.01)	0.68 (0.04–10.8)
Q5	6.28 (4.23–9.34)	1.97 (1.15–3.38)	26.2 (3.51–195)

^*^Q1 = least deprived to Q5 = most deprived

The model with overweight including obesity also yielded markedly strong associations for the outcomes {caries, no overweight or obesity} (OR Q5 vs. Q1 = 6.57; 95% CrI: 4.29 to 10.1) and coexisting caries and overweight including obesity (OR Q5 vs. Q1 = 6.97; 95% CrI: 2.84 to 16.8), and a yet weaker association for {no caries, overweight including obesity} (OR Q5 vs. Q1 = 1.31; 95% CrI: 1.00 to 1.70) (Supplementary Table S3).

## Discussion

This study addressed the geo-mapping and preventive intervention targeting approach for caries and obesity/overweight in early childhood, with the aim to inform allocation of resources to joint preventive efforts within a pediatric dental care setting. Caries and obesity, as well as caries and overweight including obesity, were analyzed combined, based on outcome data provided by all dental clinics and child health care centers in the Swedish region Kronoberg. The overall prevalences of caries, obesity, and overweight including obesity were 10.7%, 2.7%, and 12.9%, respectively, among the 8,293 preschool children studied. We found no indication of individual-level correlation between caries and obesity or overweight including obesity. Only 0.3% of the children had coexisting caries and obesity, and 1.4% had coexisting caries and overweight including obesity. Hence, we found no support for allocating resources to individually-risk-based preventive efforts on both caries and obesity/overweight within the pediatric dental care setting in the Kronoberg region. Despite these initial individual-level observations, the geo-mapping approach can still be motivated. We proceeded with analyzing geographic patterns of caries and obesity/overweight at the neighborhood-level scale, to inform allocation of resources to geographically targeted interventions. Our purpose was to identify neighborhoods where the living children show high burdens of caries and obesity/overweight – implying that resources to joint preventive efforts could be prioritized for the clinics providing dental care to children in these neighborhoods. We did not find coherent neighborhood-level associations for caries and obesity/overweight, respectively, as indicated by weaker estimated associations of neighborhood deprivation on obesity and overweight including obesity prevalences, than on caries prevalence. The geographic overlap between the 24 (out of 112) neighborhoods identified with elevated caries prevalence, on the one hand, and the 10 neighborhoods identified with elevated obesity prevalence, on the other hand, was weak; comprising 5 neighborhoods with elevated prevalence of children with coexisting caries and obesity.

The study population covered nearly 90% of the population of interest, i.e., the 3–6-year-olds who resided in the Kronoberg region at the end of 2024. Children without a previous caries assessment at a dental clinic in the Kronoberg region were not covered, consisting of those who had newly moved to this region and those who skipped dental visits. We cannot rule out that the children in the most deprived neighborhoods were slightly more underrepresented, but any selection bias should be minor. 

We observed lower prevalences of caries, obesity, and coexisting caries and obesity, compared to a study in Scotland comprising 335,361 children aged 4–7 years old, with comparable caries and obesity assessments [23]. That study reported prevalences of 30.5% (caries), 9.8% (obesity), and 3.4% (coexisting caries and obesity). Notably, calculation of the odds of obesity between the Scottish children with versus without caries yields a similar OR = 1.25, but with narrower 95% CI (1.22 to 1.28) mainly due larger sample size, than we reported herein. The Scottish study divided the study population according to neighborhood deprivation, in an analogous way as we did, and reported a pronouncedly greater odds of coexisting obesity and caries in the most deprived areas, as compared with the least deprived areas.

Our epidemiological study inferred cross-sectional associations with the caries and obesity/overweight outcomes within the 3 to 6-year-old age group, although the two health conditions were not always assessed within a short period; for 11% of the children studied, caries and BMI were assessed more than one year apart. Caries and obesity/overweight may follow temporally misaligned trajectories. Dietary patterns and early sugar exposure may first manifest as caries, while development of excess weight becomes clinically detectable later. Evidence that caries is a strong predictor of developing obesity could motivate targeted lifestyle interventions within pediatric dental care – yet given the present results. To our knowledge, there is a lack of such evidence. A 2-year longitudinal study in Finland evaluated oral health and development of excess weight based on 2,702 children aged 9–12 years at baseline, and found that caries experience alone did not associate with adiposity development [24]. Obesity and caries may follow another temporal trajectory. There are plausible biological pathways that could link excess adiposity to an elevated caries risk. Obesity has been associated with reduced stimulated salivary flow, lower salivary pH and buffering capacity, which may weaken the anti-cariogenic properties of saliva and favor demineralization of enamel [25]. Modéer et al. observed that obese adolescents had lower stimulated whole salivary flow and more decayed surfaces than normal-weight peers, supporting a pathway via hyposalivation [26]. Those findings have been further strengthened by a meta-analysis showing that obese individuals have considerably decreased salivary flow rates, although much more prominent in adolescence and adulthood than in childhood [27]. Furthermore, there is evidence that obesity is associated with pro-inflammatory cytokine and adipokine secretion, which can be reflected in saliva [28]. Such inflammatory and hormonal changes could affect both the salivary glands and immune responses in dental biofilms, thereby promoting cariogenic microbiota and caries progression [29]. However, a recent systematic review emphasized that, despite distinct salivary characteristics in individuals with obesity, the overall evidence remains limited and heterogeneous [30].

We considered a dental care setting where geographically targeted interventions could be pursued. The geo-mapping approach has been developed in several ways, since it was presented in 2011 [9]. Firstly, a more refined geographic resolution has been implemented, given by the small-area division of Sweden launched in 2018 [15]. A more accurate differential between the socioeconomic characteristics of areas (neighborhoods) studied may aid detecting underlying geographic variations in health and area-level socioeconomic relationships [31]. An appropriate neighborhood-level deprivation indicator, based on the refined geographic division of Sweden, has been suggested [16, 17]. Secondly, the computational methods for estimating Bayesian spatial models have been improved and made accessible by R-INLA [19, 32]. Thirdly, we have shown how multinomial outcome models can be estimated.

The geo-mapping approach is readily transferable to other countries where small-area divisions and sociodemographic characteristics are available. In the U.K., for instance, population data can be stratified by small areas to which indices of deprivation are available [23, 33, 34]. However, the healthcare register infrastructure for retrieving clinical outcome data with high coverage is specific to the Swedish setting.

To engage providers of pediatric dental care in lifestyle interventions addressing both oral health and obesity in childhood, there are several aspects that should be considered. We have emphasized that prioritization of such preventive resources should rely on a rational epidemiological basis. Oral health professionals’ attitudes, behaviors, and perceived barriers to prevent childhood obesity should also be regarded. A child’s weight may be seen as a medical rather than dental issue [35]. There is limited knowledge about behavior modification tools and skills that can be effectively implemented in a pediatric dental care setting to decrease risk of obesity [36].

## Conclusions

We developed the geo-mapping approach, with the purpose to identify high-risk neighborhoods of early childhood caries *and* obesity or overweight including obesity. Even though the demonstrated analysis yielded a rational basis for prioritizing resources to preventive measures focusing on oral health in early childhood [10, 37] – to providers of dental care for preschool children predominantly living in the most deprived neighborhoods – we found no support for a concurrent prioritization of more comprehensive lifestyle interventions, addressing both caries and obesity/overweight. Nevertheless*,* we acknowledge the potential for integrated child health promotion, and that dental services are universally well-positioned for early lifestyle counseling within the common risk factor framework [38].

## Supplementary Information

Supplementary Material 1.

Supplementary Material 2.

## Data Availability

The aggregated data generated and analyzed during this study can be accessed upon reasonable request from the corresponding author.

## Abbreviations

dfs: Decayed and filled surfaces
CNI: Care Need Index
BMI: Body mass index
IOTF: International Obesity Task Force
ISO-BMI: International Obesity Task Force Body Mass Index
Q1–Q5: Quintiles
OR: Odds ratio
CrI: Credible interval
CI: Confidence interval
BYM: Besag-York-Mollié
INLA: Integrated Nested Laplace Approximation

## References

[1] Twetman S, Fontana M. Patient caries risk assessment. Monogr Oral Sci. 2009;21:91–101. 19494677 10.1159/000224214

[2] Simmonds M, Llewellyn A, Owen CG, Woolacott N. Predicting adult obesity from childhood obesity: a systematic review and meta-analysis. Obes Rev. 2016;17:95–107. 26696565 10.1111/obr.12334

[3] Evensen E, Wilsgaard T, Furberg AS, Skeie G. Tracking of overweight and obesity from early childhood to adolescence in a population-based cohort - the Tromsø study, Fit Futures. BMC Pediatr. 2016;16:64. 27165270 10.1186/s12887-016-0599-5 PMC4863357

[4] Garcia RI, Kleinman D, Holt K, Battrell A, Casamassimo P, Grover J, et al. Healthy futures: engaging the oral health community in childhood obesity prevention - conference summary and recommendations. J Public Health Dent. 2017;77(Suppl 1):S136–40. 28621818 10.1111/jphd.12227

[5] Almajed OS, Aljouie AA, Alharbi MS, Alsulaimi LM. The impact of socioeconomic factors on pediatric oral health: a review. Cureus. 2024;16:e53567. 38445162 10.7759/cureus.53567 PMC10914081

[6] Sares-Jäske L, Grönqvist A, Mäki P, Tolonen H, Laatikainen T. Family socioeconomic status and childhood adiposity in Europe - a scoping review. Prev Med. 2022;160:107095. 35594926 10.1016/j.ypmed.2022.107095

[7] Manohar N, Hayen A, Fahey P, Arora A. Obesity and dental caries in early childhood: a systematic review and meta-analyses. Obes Rev. 2020;21:e12960. 31721413 10.1111/obr.12960

[8] Bakhoda MR, Haghighat Lari MM, Khosravi G, Khademi Z, Abbasi F, et al. Childhood obesity in relation to risk of dental caries: a cumulative and dose-response systematic review and meta-analysis. BMC Oral Health. 2024;24:966. 39164714 10.1186/s12903-024-04733-5 PMC11334321

[9] Strömberg U, Magnusson K, Holmén A, Twetman S. Geo-mapping of caries risk in children and adolescents - a novel approach for allocation of preventive care. BMC Oral Health. 2011;11:26. 21943023 10.1186/1472-6831-11-26 PMC3198761

[10] Holmén A, Strömberg U, Håkansson G, Twetman S. Effect of risk-based payment model on caries inequalities in preschool children assessed by geo-mapping. BMC Oral Health. 2018;18:3. 29304785 10.1186/s12903-017-0470-6 PMC5755415

[11] Aggeborn L, Öhman M. The effects of fluoride in drinking water. J Polit Econ. 2021;129:465–91.

[12] Ludvigsson JF, Bergman D, Lundgren CI, Sundquist K, Geijerstam JA, Glenngård AH, et al. The healthcare system in Sweden. Eur J Epidemiol. 2025;40:563–79. 40383868 10.1007/s10654-025-01226-9 PMC12170770

[13] Östberg AL, Kjellström AN, Petzold M. The influence of social deprivation on dental caries in Swedish children and adolescents, as measured by an index for primary health care: The Care Need Index. Community Dent Oral Epidemiol. 2017;45:233–41. 28134453 10.1111/cdoe.12281

[14] Cole TJ, Lobstein T. Extended international (IOTF) body mass index cut-offs for thinness, overweight and obesity. Pediatr Obes. 2012;7:284–94. 22715120 10.1111/j.2047-6310.2012.00064.x

[15] Statistics Sweden; Att mäta segregation på låg regional nivå. Reference no. 2017/1421 [Report in Swedish]. https://www.scb.se/contentassets/deedfb3fbe3d4abd987cfcd67dcff2e4/slutrapport-att-mata-segregation-pa-lag-regional-niva._ku2017_02404_d.pdf. Accessed 21 Aug 2025.

[16] Strömberg U, Baigi A, Holmén A, Parkes BL, Bonander C, Piel FB. A comparison of small-area deprivation indicators for public-health surveillance in Sweden. Scand J Public Health. 2023;51:520–6. 34282665 10.1177/14034948211030353 PMC10259086

[17] van der Velde L, Shabaan AN, Mattsson M, Bodin T, Eikemo TA, Swartling Peterson S, et al. An index of multiple deprivation in Sweden: measuring area-level socio-economic inequalities. Eur J Public Health. 2025;35:ckaf138. 10.1093/eurpub/ckaf138 PMC12707479 40882978

[18] Riebler A, Sørbye SH, Simpson D, Rue H. An intuitive Bayesian spatial model for disease mapping that accounts for scaling. Stat Methods Med Res. 2016;25:1145–65. 27566770 10.1177/0962280216660421

[19] Lindgren F, Rue H. Bayesian spatial modelling with R-INLA. J Stat Softw. 2015;63:1-.

[20] Baker SG. The multinomial-poisson transformation. J R Stat Soc Ser Stat. 1994;43:495–504.

[21] Richardson S, Thomson A, Best N, Elliott P. Interpreting posterior relative risk estimates in disease-mapping studies. Environ Health Perspect. 2004;112:1016–25. 15198922 10.1289/ehp.6740 PMC1247195

[22] Kim I, Kang HY, Khang YH. Comparison of Bayesian spatiotemporal models for small-area life expectancy: a simulation study. Am J Epidemiol. 2023;192:1396–405. 36963380 10.1093/aje/kwad073

[23] Stewart R, Conway DI, Macpherson LMD, Sherriff A. Obesity and dental caries in childhood: trends in prevalence and socioeconomic inequalities-a multicohort population-wide data linkage study. Arch Dis Child. 2024;109:642–8. 38724064 10.1136/archdischild-2023-326587 PMC11789570

[24] Lommi S, Leinonen J, Pussinen P, Furuholm J, Kolho KL, Viljakainen H. Burden of oral diseases predicts development of excess weight in early adolescence: a 2-year longitudinal study. Eur J Pediatr. 2024;183:4093–101. 38960905 10.1007/s00431-024-05663-8 PMC11322208

[25] Lynge Pedersen AM, Belstrøm D. The role of natural salivary defences in maintaining a healthy oral microbiota. J Dent. 2019;80(Suppl 1):S3–12. 30696553 10.1016/j.jdent.2018.08.010

[26] Modéer T, Blomberg CC, Wondimu B, Julihn A, Marcus C. Association between obesity, flow rate of whole saliva, and dental caries in adolescents. Obesity (Silver Spring). 2010;18:2367–73. 20339364 10.1038/oby.2010.63

[27] Hatipoglu O, Maras E, Hatipoglu FP, Saygin AG. Salivary flow rate, pH, and buffer capacity in the individuals with obesity and overweight; a meta-analysis. Niger J Clin Pract. 2022;25:1126–42. 35859475 10.4103/njcp.njcp_1760_21

[28] Zyśk B, Ostrowska L, Smarkusz-Zarzecka J. Salivary adipokine and cytokine levels as potential markers for the development of obesity and metabolic disorders. Int J Mol Sci. 2021;22:11703. 34769133 10.3390/ijms222111703 PMC8584047

[29] Roa I, Del Sol M. Obesity, salivary glands and oral pathology. Colomb Med (Cali). 2018;49:280–7. 30700921 10.25100/cm.v49i3.3919 PMC6342082

[30] Deng Q, Wong HM, Peng S. Alterations in salivary profile in individuals with dental caries and/or obesity: a systematic review and meta-analysis. J Dent. 2024;151:105451. 39505293 10.1016/j.jdent.2024.105451

[31] Piel FB, Fecht D, Hodgson S, Blangiardo M, Toledano M, Hansell AL, et al. Small-area methods for investigation of environment and health. Int J Epidemiol. 2020;49:686–99. 32182344 10.1093/ije/dyaa006 PMC7266556

[32] Blangiardo M, Cameletti M. Spatial and spatio-temporal Bayesian models with R-INLA. New York: Wiley; 2015.

[33] Office for National Statistics. Census 2021 geographies. https://www.ons.gov.uk/methodology/geography/ukgeographies/censusgeographies/census2021geographies. Accessed 27 Aug 2025.

[34] The English Indices of Deprivation 2019 – LSOA level. https://opendatacommunities.org/resource?uri=http%3A%2F%2Fopendatacommunities.org%2Fdata%2Fsocietal-wellbeing%2Fimdh2019%2Findices. Accessed 27 Aug 2025.

[35] Wright R, Casamassimo PS. Assessing attitudes and actions of pediatric dentists toward childhood obesity and sugar-sweetened beverages. J Public Health Dent. 2017;77(Suppl 1):S79–87. 28712110 10.1111/jphd.12240

[36] Mallonee LF, Boyd LD, Stegeman C. A scoping review of skills and tools oral health professionals need to engage children and parents in dietary changes to prevent childhood obesity and consumption of sugar-sweetened beverages. J Public Health Dent. 2017;77(Suppl 1):S128–35. 28742239 10.1111/jphd.12237

[37] Anil S, Anand PS. Early childhood caries: prevalence, risk factors, and prevention. Front Pediatr. 2017;5:157. 28770188 10.3389/fped.2017.00157 PMC5514393

[38] Sheiham A, Watt RG. The common risk factor approach: a rational basis for promoting oral health. Community Dent Oral Epidemiol. 2000;28:399–406. 11106011 10.1034/j.1600-0528.2000.028006399.x

