# Supplementary material for: Geo-mapping of caries and obesity in preschool children: a Swedish register-based study
Source: BMC Oral Health. 2026 Jan 30;26:370. doi: 10.1186/s12903-026-07783-z (PMC12930677; doi:10.1186/s12903-026-07783-z)
Supplement: Supplementary file 1 — Supplementary Material 1. [file 12903_2026_7783_MOESM1_ESM.pdf]

Table S1. Description of the study population and the outcomes of caries and overweight including obesity.

		No. of children	Children with outcome {no caries, no overweight or obesity}, n (%)	Children with outcome {caries, no overweight or obesity}, n (%)	Children with outcome {no caries, overweight including obesity}, n (%)	Children with outcome {caries, overweight including obesity}, n (%)
	Study population	8,293	6,448 (77.8%)	775 (9.3%)	957 (11.5%)	113 (1.4%)
Sex	Boys	4,278	3,381 (79.0%)	414 (9.7%)	430 (10.1%)	53 (1.2%)
	Girls	4,015	3,067 (76.4%)	361 (9.0%)	527 (13.1%)	60 (1.5%)
Age (years)	6	2,217	1,582 (71.4%)	342 (15.4%)	246 (11.1%)	47 (2.1%)
	5	2,295	1,768 (77.0%)	241 (10.5%)	251 (10.9%)	35 (1.5%)
	3–4	3,781	3,098 (81.9%)	192 (5.1%)	460 (12.2%)	31 (0.8%)

Table S2. Prevalences of caries and overweight including obesity in groups of children according to neighborhood deprivation.

Neighborhood deprivation* (number of neighborhoods)	No. of children	Children with outcome {no caries, no overweight or obesity}, n (%)	Children with outcome {caries, no overweight or obesity}, n (%)	Children with outcome {no caries, overweight including obesity}, n (%)	Children with outcome {caries, overweight including obesity}, n (%)
Q1 (13)	1,220	1,036 (84.9%)	46 (3.8%)	132 (10.8%)	6 (0.5%)
Q2 (17)	1,373	1,147 (83.5%)	64 (4.7%)	158 (11.5%)	4 (0.3%)
Q3 (24)	1,786	1,437 (80.5%)	103 (5.8%)	226 (12.7%)	20 (1.1%)
Q4 (30)	1,921	1,480 (77.0%)	194 (10.1%)	213 (11.1%)	34 (1.8%)
Q5* (28)	1,993	1,348 (67.6%)	368 (18.5%)	228 (11.4%)	49 (2.5%)

* Q1 = least deprived to Q5 = most deprived.

Table S3. Estimated associations of caries and overweight including obesity with neighborhood deprivation.

Neighborhood deprivation*	OR (95% CrI) for outcome {caries, no overweight or obesity}	OR (95% CrI) for outcome {no caries, overweight including obesity}	OR (95% CrI) for outcome {caries, overweight including obesity}
Q1	1.00 (reference)	1.00 (reference)	1.00 (reference)
Q2	1.28 (0.79–2.10)	1.06 (0.80–1.40)	0.59 (0.16–2.12)
Q3	1.76 (1.11–2.79)	1.21 (0.92–1.57)	2.62 (1.02–6.73)
Q4	3.10 (2.01–4.80)	1.09 (0.84–1.42)	4.27 (1.74–10.5)
Q5	6.57 (4.28–10.1)	1.31 (1.00–1.70)	6.97 (2.89–16.8)

* Q1 = least deprived to Q5 = most deprived.
