# Supplementary material for: Geo-mapping of caries and obesity in preschool children: a Swedish register-based study
Source: BMC Oral Health. 2026 Jan 30;26:370. doi: 10.1186/s12903-026-07783-z (PMC12930677; doi:10.1186/s12903-026-07783-z)

**Supplementary Figure S1.** Observed neighborhood-level prevalences vs. posterior predictive medians along with 95% credible intervals (CrIs) for each specific adverse outcome – {caries, no obesity} (**A**), {no caries, obesity} (**B**), and {caries, obesity} (**C**). The results show high predictive performance for {caries, no obesity}, where observed prevalences are relatively common; the predicted medians closely follow the dashed line of equality, and the 95% CrIs cover the this line for 94% of the 112 neighborhoods. For {no caries, obesity} and {caries, obesity}, the coverage proportions are lower (63% and 14%, respectively), which should be expected for rare outcomes that have been spatially smoothed – the lower coverage proportions reflect data sparsity rather than model misspecification

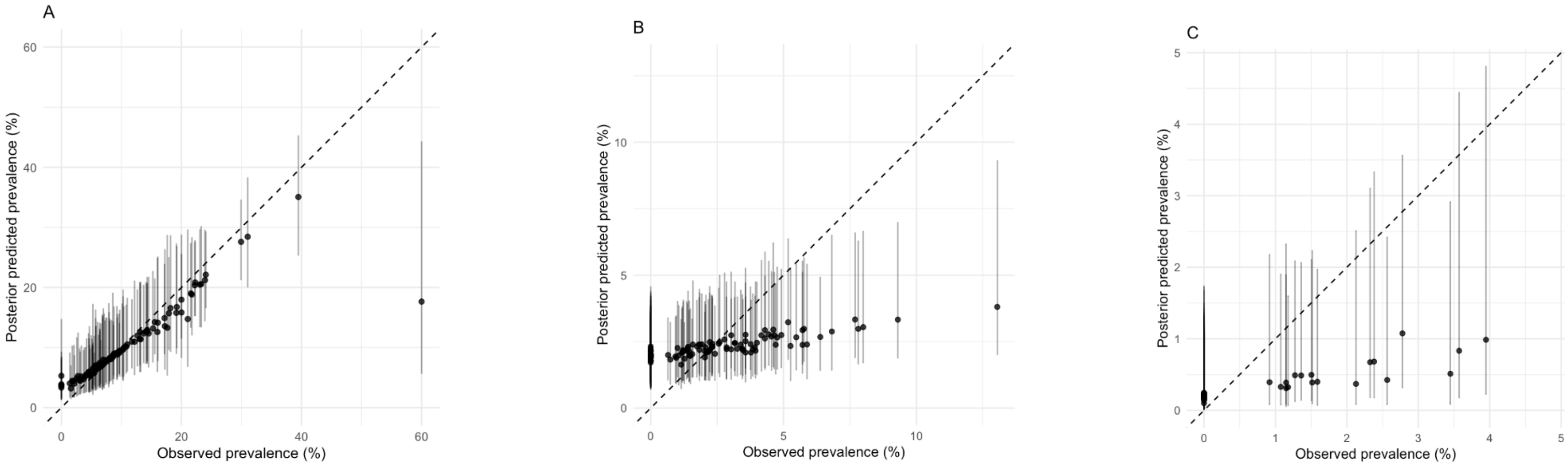

Supplement: Supplementary file 2 — Supplementary Material 2. [file 12903_2026_7783_MOESM2_ESM.pdf]
